# Glutamine and glutamate supplementation in sow diets during late gestation and lactation reduces farrowing duration, improves colostrum amino acid content, and enhances piglet weaning weight

**DOI:** 10.1093/jas/skaf367

**Published:** 2025-10-22

**Authors:** Maria Vitória S Sousa, Charles Kiefer, Lais Fernanda L Reis, Ana Gabrielli dos Santos Fagundes Euzebio, Luiza O Possa, Sung Woo Kim, Gabriel C Rocha

**Affiliations:** Department of Animal Science Post-Graduation Program, Universidade Federal de Mato Grosso do Sul, Faculdade de Medicina Veterinária e Zootecnia, Campo Grande, Brazil; Department of Animal Science Post-Graduation Program, Universidade Federal de Mato Grosso do Sul, Faculdade de Medicina Veterinária e Zootecnia, Campo Grande, Brazil; Muscle Biology and Nutrigenomics Laboratory, Department of Animal Science, Universidade Federal de Viçosa, Minas Gerais, Brazil; Department of Animal Science Post-Graduation Program, Universidade Federal de Mato Grosso do Sul, Faculdade de Medicina Veterinária e Zootecnia, Campo Grande, Brazil; Laboratory of Molecular Biotechnology, Department of Biochemistry and Molecular Biology, Universidade Federal de Viçosa, Viçosa, Brazil; Department of Animal Science, North Carolina State University, Raleigh, NC; Muscle Biology and Nutrigenomics Laboratory, Department of Animal Science, Universidade Federal de Viçosa, Minas Gerais, Brazil

**Keywords:** Colostrum, gestation, glutamate, glutamine, lactation, piglet performance

## Abstract

Functional amino acids including glutamine and glutamate (Gln/Glu) play critical roles in supporting intestinal health, antioxidant defense, and metabolic regulation during periods of increased physiological demand, including late gestation and lactation in sows. This study evaluated the effects of Gln/Glu supplementation in sow diets during late gestation and lactation on sow performance, reproductive parameters, colostrum composition, blood biomarkers, and fecal microbiota. A total of 43 DanBred sows (parity 4.0 ± 1.7; body weight 270 ± 31 kg) were allocated using a randomized complete block design with parity and body weight as blocks and fed either a control diet or a Gln/Glu-supplemented diet (10 g/kg) from day 86 of gestation until weaning (day 21 of lactation). Sows fed the Gln/Glu-supplemented diet exhibited reduced farrowing duration (*P *< 0.05). Colostrum from sows fed the Gln/Glu-supplemented diet showed higher concentrations of methionine, cystine, isoleucine, leucine, alanine, and tyrosine compared to the control group (*P *< 0.05), with trends for increased levels of histidine (*P *= 0.083), glutamic acid (*P *= 0.081), glycine (*P *= 0.062), and serine (*P *= 0.089). A trend toward higher average daily feed intake during lactation (*P *= 0.089) and increased insulin concentration (*P *= 0.077) was also observed. Piglet weaning weight was improved (P < 0.05) in sows fed the Gln/Glu-supplemented diet. No effects were observed on gestation weight gain, lactation weight loss, backfat thickness, body condition score, or most blood biochemical and immunological parameters. The fecal bacterial load of *Escherichia coli* and *Clostridium perfringens* was unaffected by treatment. These findings suggest that supplementing sow diets with Gln/Glu during late gestation and lactation may enhance farrowing efficiency, improve colostrum amino acid content, and improve neonatal growth during lactation.

## Teaser

Glutamine and glutamate reduced sow farrowing duration, enhanced colostrum amino acid concentrations, and improved piglet weight at weaning. This suggests a promising strategy to optimize sow and litter performance during late gestation and lactation.

## Introduction

The peripartum period for sows is characterized by substantial physiological and metabolic demands, including fetal development, colostrogenesis, farrowing, and lactation (Kim et al., 2013). These processes require increased nutrient intake, particularly amino acids (AA), to support maternal health, neonatal development, and reproductive efficiency (Watford, 2015; Zhao and Kim, 2020). Functional amino acids such as glutamine and glutamate have been shown to play critical roles in energy metabolism, antioxidant defense, immune function, and nitrogen transport (Newsholme et al., 2003; Kim et al., 2006; Wu, 2009). Their supplementation during late gestation and lactation may offer metabolic and physiological support to the sow and offspring during this high-demand period.

Glutamine and glutamate represent the most abundant AA in sow colostrum and milk (Wu and Knabe, 1994; Li et al., 2009; Rezaei et al., 2022) playing pivotal roles in supporting neonatal intestinal development, immune system maturation, and as primary substrates for rapidly proliferating cells such as enterocytes and lymphocytes (Stoll et al., 1999; Correia et al., 2023). In addition, these actions are particularly relevant during lactation, a phase for the sow marked by elevated nutrient demands and systemic stress, and could reduce systemic inflammation and improve gastrointestinal health (Duan et al., 2016), thereby creating conditions favorable for greater feed intake during lactation. Furthermore, glutamate contributes to energy metabolism via the tricarboxylic acid (TCA) cycle and may influence endocrine responses such as insulin secretion (Mateo et al., 2007; Yang et al., 2014). Insulin, in turn, is a critical regulator of nutrient utilization and mammary gland metabolism during lactation (Boyd et al., 1995; Holen et al., 2022), suggesting that Gln/Glu-mediated modulation of insulin could support improved metabolic efficiency and sow performance (Koketsu et al., 1998; Rezaei et al., 2016).

Moreover, evidence suggests that glutamine and glutamate may support milk synthesis by enhancing AA availability and activating intracellular signaling pathways such as mTOR in mammary epithelial cells (Li et al., 2009; Wang et al., 2018). Their metabolic interrelationships with essential AA, such as branched-chain amino acids (BCAA), may further influence colostrum composition, which is essential for neonatal immunity, growth, and thermoregulation (Li et al., 2009; Gondret et al., 2021; Rezaei et al., 2022).

Despite growing interest in functional AA nutrition, limited research has examined the effects of combined glutamine and glutamate (Gln/Glu) supplementation in sow diets during the late gestation–lactation continuum. The present study was designed to evaluate the effects of dietary Gln/Glu supplementation from d 86 of gestation to weaning on sow performance, reproductive outcomes, colostrum composition (including AA content), blood biochemical and immunological parameters, and fecal microbiota composition.

## Material and Methods

All methods involving the handling of pigs followed the ethical principles of animal research (CONCEA) and were approved by the Commission of Ethics in the Use of Production Animals (CEUA), protocol 1359/2025.

### Experimental design

On d 86 of gestation, 43 sows [PIC 337 (Large White × Landrace × Duroc × Pietrain) × DB 90 (Large White × Landrace)] were assigned to dietary treatments based on a randomized complete block design using parity (4.0 ± 1.7) and body weight (269.2 ± 30.5 kg) as blocking factors. Sows were allotted to one of two dietary treatments from d 86 of gestation through lactation: a control diet or a supplemented diet where 10 g/kg of a glutamine and glutamate mixture was added on top of the control diet (Gln/Glu; commercial mixture guaranteed to contain minimum 10% L-glutamine and minimum 10% L-glutamate). A total of 21 and 22 sows, which served as the experimental unit, were assigned to the control and Gln/Glu groups, respectively. The gestation and lactation control diets (Table 1) were formulated to meet or exceed the nutrient requirements for sows with a body weight of 280 kg during late gestation and 260 kg during lactation, respectively, as per the Brazilian Tables for Poultry and Swine (Rostagno et al., 2017).

**Table 1.** Ingredient and calculate composition of experimental diets (as-fed basis)

Ingredient, g/kg	Gestation	Lactation
** Sorghum**	774.0	-
** Corn**	-	571.5
** Soybean meal**	177.0	259.0
** Soybean oil**	-	45.0
** Biscuit meal**	-	40.0
** Meat and bone meal**	25.0	25.0
** Sugar**	-	25.0
** Dicalcium phosphate**	5.0	8.5
** Limestone**	7.5	6.8
** Salt**	5.0	4.0
** Sodium bicarbonate**	-	4.0
** Gestation premix^1^**	4.0	-
** Lactation premix^2^**	-	4.0
** L-Lysine HCl**	1.5	3.5
** Mycotoxin binder**	1.0	1.0
** L-Threonine**	-	1.2
** DL-Methionine**	0.2	0.9
** L-Tryptophan**	-	0.6
** Total**	1,000	1,000
**Calculate composition**		
** Metabolizable energy, kcal/kg**	3,150	3,550
** Crude protein, %**	15.4	19.7
** SID^3^ lysine, %**	0.73	1.15
** SID methionine + cystine, %**	0.46	0.70
** SID methionine, %**	0.25	0.31
** SID threonine, %**	0.52	0.80
** SID tryptophan, %**	0.17	0.23
** SID valine, %**	0.60	0.98
** SID glutamine + glutamate, %**	2.79	2.85
** STTD^4^ phosphorus, %**	0.45	0.50
** Calcium, %**	0.90	0.80
** Sodium, %**	0.20	0.20

1 Gestation premix included: cobalt carbonate (50,000 mg/kg), copper sulfate (18,750 mg/kg), iron sulfate (25,000 mg/kg), calcium iodate (300,000 mg/kg), manganese sulfate (12,500 mg/kg), sodium selenite (100,000 mg/kg), hydroxy analogue of selenomethionine, zinc oxide (50,000 g/kg), vitamin A (3,000,000 IU/kg), vitamin D3 (625,000 IU/kg), vitamin E (21,250 IU/kg), vitamin K3 (626 mg/kg), vitamin B1 (550 mg/kg), vitamin B2 (2,000 mg/kg), vitamin B6 (750 mg/kg), vitamin B12 (7,500 mcg/kg), folic acid (750 mg/kg), calcium pantothenate (5,000 mg/kg), biotin (150 mg/kg), biocoline (20,000 g/kg), niacin (7,500 mg/kg), enzymatic additive, diatomite, BHT (24,500 g/kg), Kaolin, organic selenium (12,500 mg/kg), total selenium (100,000 mg/kg), 6-phytase (125,000 IU/kg).

2 Lactation premix included: cobalt carbonate (50,000 mg/kg), copper sulfate (25,000 mg/kg), iron sulfate (25,000 mg/kg), calcium iodate (300,000 mg/kg), manganese sulfate (12,500 g/kg), sodium selenite (100,000 mg/kg), hydroxy analogue of selenomethionine, zinc oxide (50,000 g/kg), vitamin A (3,750,000 IU/kg), vitamin D3 (781,250 IU/kg), vitamin E (21,875 IU/kg), vitamin K3 (1,250 mg/kg), vitamin B1 (687.5 mg/kg), vitamin B2 (2,500 mg/kg), vitamin B6 (937.5 mg/kg), vitamin B12 (9,375 mcg/kg), folic acid (937.5 mg/kg), calcium pantothenate (6,250 mg/kg), biotin (150 mg/kg), choline chloride (168,840 g/kg), niacin (9,375 mg/kg), red fruit flavor, enzymatic additive, diatomite, BHT (49,000 g/kg), kaolin, Endo-1,4-Beta xylanase (372,000 U/kg), 6-Phytase (125,000 U/kg).

3 SID, Standardized ileal digestible.

4 STTD, Standardized total tract digestible.

### Housing, feeding, and management

From d 86 to d 108 of gestation, sows were housed in individual stalls (2.2 × 0.7 m) and received 2.75 kg/d of gestation diet. On d 108 of gestation, sows were moved to the farrowing facility and housed in individual farrowing crates (2.4 × 1.8 m) equipped with plastic flooring, individual feed troughs, and drinking nipples. From d 108 until farrowing, sows were fed 3.0 kg/d of lactation diet. Feed was withheld on the day of farrowing, and from d 2 postpartum until weaning (d 21 of lactation), sows were fed *ad libitum*, with refusals weighed daily to calculate feed intake. Farrowing was induced on d 114 of gestation using cloprostenol sodium (0.8 mL, Sincrocio, Ouro Fino, Cravinhos, São Paulo, Brazil).

During the first 48 h postpartum, the original litter was maintained with the sow. After 48 h, litters were standardized to 13 piglets per sow by cross-fostering within the same treatment. Surplus piglets were allocated to nurse sows according to standard farm practice, a procedure that also helped reduce experimental variability by minimizing the influence of extreme litter sizes and allowing a more reliable evaluation of dietary treatment effects. Throughout lactation, piglets did not have access to creep feed. Piglets were ear tagged at birth, teeth clipped within 24 h postpartum, and tails docked on d 3 postpartum. All piglets received an intramuscular iron injection on d 5 postpartum, and males were left intact. An infrared heat lamp was provided in the creep area for piglet thermal comfort.

During the experimental period, the average air temperatures were 27.6 °C and 28.7 °C during gestation and lactation, respectively. The relative humidity was 65.5% and 70.0% during gestation and lactation, respectively.

### Performance and reproductive analyses

At d 86 of gestation, d 108 (entry to the farrowing unit), and at weaning, sows were weighed using a digital livestock scale (MGRcampo, Toledo do Brasil, SP, Brazil). Backfat thickness was measured via ultrasound (MTU-100 backfat meter, Microem, SP, Brazil) at the P2 position (6.5 cm off the midline at the level of the last rib). Body condition score was assessed using a caliper (PIC North America, TN, USA).

Farrowing performance were recorded, including farrowing duration, the interval between the birth of consecutive piglets, and the occurrence of dystocic farrowings. A farrowing was considered dystocic when manual obstetrical assistance was required. Intervention was performed only when the interval after the birth of the previous piglet exceeded 30 minutes and after non-invasive measures, such as abdominal massage or encouraging the sow to stand or change position, had been attempted without success. Reproductive outcomes included total piglets born, liveborn piglets, stillborns, mummified, and body weight at birth, after cross fostering, and weaning. Litter weight uniformity at birth was assessed based on the proportion of piglets weighing <1.0 kg, between 1.0 and 1.5 kg, and >1.5 kg, adapted from Feldpausch et al. (2019).

Daily feed intake during lactation and wean-to-estrus interval, were measured. Daily sow milk yield (kg/d) was estimated using the equation described by Noblet and Etienne (1989): (2.50 × ADG + 80.2 × BWi + 7) × litter size/1000, where ADG is the piglet average daily gain (g/d), BWi is weight after cross fostering (g), and litter size is the number of piglets suckling after cross-fostering.

### Colostrum sampling and analyses

Colostrum samples (40 mL) were collected from all sows on the day of parturition, within 6 h of the birth of the first piglet, without administration of oxytocin. The samples were obtained from the first five anterior pairs of functional teats, and one composite sample was taken per sow. The milking procedure lasted between 10 and 20 min, depending on the sow. Samples were identified and stored at −80°C for analysis of nutrient concentrations and AA.

The frozen colostrum samples were thawed at 4 °C and analyzed for dry matter, fat, crude protein, and lactose concentrations. All analyses were performed in duplicate. Dry matter was determined by oven-drying at 105 °C until constant weight (AOAC, 2005; Method 923.23). Fat content was determined using the Gerber method according to AOAC (2005; Method 989.05). Crude protein concentration was determined by the Kjeldahl method, following AOAC (2005; Method 991.20), based on nitrogen determination and the use of a 6.38 conversion factor for milk protein. Lactose concentration was analyzed using the Lane-Eynon method (Fehling’s solution), as described by AOAC (2005; Method 922.09), which is based on the titration of reducing sugars under standard conditions.

Total AA concentrations in colostrum were determined by high-performance liquid chromatography (HPLC) with ion-exchange chromatography and photometric detection after post-column derivatization with ninhydrin. Samples were hydrolyzed with 6 M hydrochloric acid (HCl) at 110 °C for 24 h in sealed tubes under a nitrogen atmosphere. Methionine and cystine were stabilized by oxidation with performic acid prior to hydrolysis. The analytical procedure followed the methodology described in the Compêndio Brasileiro de Alimentação Animal (Sindirações, 2023). Tryptophan was not determined due to degradation under acid hydrolysis conditions. All analyses were performed in duplicate. Results were expressed as g/100 g of colostrum and are presented in the manuscript as percentages (%).

### Blood sampling and analyses

One day before weaning, after 12 h fasting, blood samples were collected from all sows via puncture of the auricular vein into 10 mL uncoated vacuum and were centrifuged (3,500 × g for 10 min, Daiki, 80-2BDM, Brazil) to separate the serum. The following parameters were determined: glucose (mg/dL; enzymatic colorimetric method, Kovalent, BS-380 analyzer, Mindray, Shenzhen, China), serum urea nitrogen concentration (mg/dL; Linklab, software PNCQ, Ureal Cobas C311, Roche Diagnostics, Indianapolis, IN, USA), creatinine (mg/dL; kinetic method, Kovalent, BS-380, Mindray, Shenzhen, China), immunoglobulin G (IgG) (mg/dL; Atellica CH IgG_2, Siemens Healthineers, Erlangen, Germany), immunoglobulin A (IgA) (mg/dL; 9D98-21 Reagent Kit, Architect cSystems, Abbott Laboratories, Chicago, IL, USA), insulin-like growth factor I (IGF-1) (ng/ml; IMMULITE, Siemens, Malvern, PA, USA), and insulin (µUI/ml; Atellica IM, Attelica - IM Analyzer, Siemens, Malvern, PA, USA).

### Fecal bacterial analyses

One day before weaning, fecal samples were collected from all sows using rectal swabs and stored at −80°C until processing. Total genomic DNA was extracted from the samples using the E.Z.N.A. Stool DNA Kit (D4015-02, Omega, Inc., USA) following the manufacturer’s instructions. The extracted DNA was eluted in 40 μL of elution buffer and stored at −20°C until quantification. DNA concentration was measured using a UV/Vis microplate spectrophotometer (Multiskan SkyHigh Microplate Spectrophotometer—Thermo Scientific).

The bacterial load of *Escherichia coli*, *Clostridium perfringens*, and *Salmonella typhimurium* in fecal samples was determined using quantitative PCR (qPCR). Reactions were performed on a StepOn Real-Time PCR System (Applied Biosystems) using GoTaq qPCR Master Mix (Promega, Madison, WI, USA). Each 10 μL reaction mixture contained 5 μL of GoTaq qPCR Master Mix, 0.1 μL of CXR Reference Dye, 0.2 μM of each primer (forward and reverse), and 100 ng of total genomic DNA extracted from feces.

The primers used were: E. coli: ECForward (5′-TGATTGGCAAAATCTGGCCG-3′) and ECReverse (5′-CGAAATCGCCCAAATCGCCAT-3′) C. perfringens: CPForward (5′-AAATGTAACAGCAGGGGCA-3′) and CPReverse (5′-TGAAATTGCAGCAACTCTAGC-3′) S. typhimurium: STForward (5′-GCTGCTTTCTCTACTTAAC-3′) and STReverse (5′-GTAATGGAATGACGAACAT-3′). The thermal cycling conditions included an initial denaturation at 95 °C for 10 minutes, followed by 40 cycles of denaturation at 95 °C for 30 seconds and annealing at 60 °C for 1 minute. Amplification curves were generated, and a dissociation curve was produced to confirm specificity. The melting curve analysis was conducted with an initial denaturation at 95 °C for 30 seconds, annealing at 60 °C for 1 minute, followed by a temperature increase of 1 °C every 30 seconds until reaching 95 °C. All qPCR reactions were performed in triplicate. Negative controls consisted of triplicate reactions without template DNA.

Amplification curves and Ct (cycle threshold) values were recorded and analyzed using StepOne v2.1 software. Data visualization and statistical analysis were performed using GraphPad Prism 9.0.

### Statistical analyses

All data were analyzed using the General Linear Model (GLM) procedure of SAS (SAS Inst. Inc., Cary, NC). The statistical model included dietary treatment as the fixed effect, and sow served as the experimental unit for all variables. Parity and body weight at d 86 of gestation were random effects used as blocking factors in the randomized complete block design. Prior to analysis, data were assessed for normality and homogeneity of variances using the Shapiro–Wilk and Levene’s tests, respectively. Treatment means were compared using least squares means, and significance was declared at *P *< 0.05. Values between *P *≥ 0.05 and *P *< 0.10 were considered trends. Results are presented as least squares means with their associated standard errors.

## Results

There were no effects (*P *> 0.10) of Gln/Glu supplementation on sow body weight at any time point during gestation or lactation (Table 2). Similarly, gestation weight gain and lactation weight loss were not affected (*P *> 0.10). Average daily feed intake (ADFI) during lactation showed a tendency (*P *= 0.089) to increase in the Gln/Glu group. Backfat thickness and body condition scores were not affected (*P *> 0.10) throughout the experimental period. Estimated daily milk production and wean-to-estrus interval also did not differ between treatments (*P *> 0.10).

**Table 2.** Performance of sows fed either a control diet or a diet supplemented with glutamine and glutamate (Gln/Glu) from d 86 of gestation to d 21 of lactation

Item	Control	Gln/Glu	SEM	*P*-Value
**Number of sows**	21 ± 1.7	22 ± 1.7	-	-
**Body weight, kg**				
** Day 86 of gestation**	270	269	5	0.873
** Day 108 of gestation**	295	290	5	0.446
** Gestation weight gain**	24	22	1	0.353
** Sow weaning weight**	259	255	5	0.460
** Lactation weight loss**	35	37	2	0.765
**Lactation ADFI^1^, kg**	5.35	5.86	0.18	0.089
**Backfat thickness, mm**				
** Day 86 of gestation**	16.5	15.3	0.5	0.167
** Day 108 of gestation**	16.9	15.5	0.5	0.124
** Gestation gain**	0.4	0.2	0.1	0.255
** Weaning**	16.1	15.2	0.4	0.377
** Lactation loss**	0.8	0.3	0.2	0.755
**Body condition score^2^**				
** Day 86 of gestation**	18.6	18.0	0.2	0.300
** Day 108 of gestation**	18.4	18.2	0.2	0.614
** Weaning**	17.1	16.5	0.3	0.399
**Daily milk yield^3^, kg**	7.2	7.7	0.3	0.101
**Wean to estrus interval, days**	3.9	4.1	0.6	0.335

1 ADFI, average daily feed intake.

2 Caliper (PIC North America, TN, USA).

3 Estimated based on Noblet and Etienne (1989).

Sows fed the Gln/Glu diet exhibited reduced farrowing time (*P *< 0.05) and shorter intervals between birth of piglets (*P *< 0.05) compared to the control group (Table 3). The number of dystocic farrowings was not affected (*P *> 0.10) by dietary treatment.

**Table 3.** Farrowing characteristics of sows fed either a control diet or a diet supplemented with glutamine and glutamate (Gln/Glu) from d 86 of gestation to d 21 of lactation

Item	Control	Gln/Glu	SEM	*P*-Value
**Farrowing duration, min**	309	230	19	0.031
**Piglet interval, min**	24.0	13.3	2.35	0.023
**Dystocic farrowings, n**	0.21	0.19	0.06	0.864

Sows receiving the Gln/Glu diet had increased total born (*P *< 0.05) and liveborn piglets and daily weight gain at weaning (*P *< 0.05), along with greater litter weight at birth (*P *< 0.05) (Table 4). Piglet body weight at weaning was increased in the Gln/Glu group (*P *< 0.05). No effects (*P *> 0.10) were observed on the number of stillborn or mummified piglets or body weight at birth. Additionally, weaning age and litter size were not influenced (*P *> 010) by dietary treatment.

**Table 4.** Performance of litters from sows fed either a control diet or a diet supplemented with glutamine and glutamate (Gln/Glu) from d 86 of gestation to d 21 of lactation

Item	Control	Gln/Glu	SEM	*P*-Value
**Total born, n**	15.6	18.1	0.72	0.034
**Liveborn, n**	13.8	16.7	0.74	0.043
**Stillborn, n**	1.21	1.09	0.38	0.099
**Mummified, n**	0.52	0.27	0.12	0.279
**Liveborn body weight at birth, kg**	1.27	1.24	0.04	0.736
**Litter weight at birth, kg**	18.2	20.4	3.23	0.039
**Litter size after cross fostering^1^, n**	13.0	13.0	0.1	0.888
**Body weight after cross fostering, kg**	1.42	1.45	0.04	0.570
**Weaning age, days**	19.5	19.5	0.3	0.646
**Litter size at weaning, n**	12.2	12.5	0.1	0.288
**Body weight at weaning, kg**	4.44	4.76	0.10	0.020
**Daily weight gain, g**	172	189	6	0.029

1 After 48 h, litters were standardized to 13 piglets per sow by cross-fostering within the same treatment.

The distribution of piglet birth weights was not affected (*P *> 0.10) by Gln/Glu supplementation (Table 5). Colostrum dry matter, ether extract, lactose, and crude protein contents were not affected (*P *> 0.10) by Gln/Glu supplementation (Table 6).

**Table 5.** Birth weight by various weight groups from sows fed either a control diet or a diet supplemented with glutamine and glutamate (Gln/Glu) from d 86 of gestation to d 21 of lactation

Item, %	Control	Gln/Glu	SEM	*P*-Value
**<1.0 kg**	15.3	14.4	0.3	0.841
**1.0 to 1.5 kg**	54.9	54.2	0.7	0.342
**>1.5 kg**	29.9	31.4	0.4	0.327

**Table 6.** Composition in colostrum from sows fed either a control diet or a diet supplemented with glutamine and glutamate (Gln/Glu) from d 86 of gestation to d 21 of lactation

Item^1^, %	Control	Gln/Glu	SEM	*P*-Value
Dry matter	24.37	24.51	0.32	0.829
Ether extract	7.74	7.60	0.15	0.658
Lactose	3.87	3.72	0.13	0.573
Crude protein	16.25	17.62	0.46	0.103

1 Colostrum collected on the day of parturition, within 6 h of the birth of the first piglet.

Colostrum concentrations of methionine, cystine, isoleucine, leucine, alanine, and tyrosine were greater (*P *< 0.05) in sows fed the Gln/Glu diet (Table 7). Additionally, there were tendencies for higher concentrations of glutamic acid (*P *= 0.081), glycine (*P *= 0.062), histidine (*P *= 0.083), and serine (*P *= 0.089) in the Gln/Glu group. No differences were observed for lysine, threonine, arginine, aspartic acid, phenylalanine, or valine (*P *> 0.10).

**Table 7.** Amino acid composition in colostrum from sows fed either a control diet or a diet supplemented with glutamine and glutamate (Gln/Glu) from d 86 of gestation to d 21 of lactation

Item^1^, %	Control	Gln/Glu	SEM	*P*-Value
**Arginine**	0.81	0.85	0.07	0.386
**Histidine**	0.40	0.43	0.01	0.083
**Isoleucine**	0.54	0.60	0.01	0.033
**Leucine**	1.48	1.64	0.07	0.025
**Lysine**	1.10	1.12	0.09	0.130
**Methionine**	0.22	0.24	0.01	0.048
**Phenylalanine**	0.72	0.77	0.02	0.218
**Threonine**	0.95	1.05	0.09	0.107
**Valine**	1.06	1.10	0.09	0.432
**Indispensable amino acids**	7.28	7.80	0.15	0.046
**Alanine**	0.70	0.78	0.02	0.008
**Aspartic Acid**	1.30	1.36	0.07	0.327
**Cystine**	0.27	0.30	0.01	0.042
**Glutamic Acid**	2.47	2.66	0.07	0.081
**Glycine**	0.56	0.61	0.02	0.062
**Serine**	1.05	1.15	0.09	0.089
**Tyrosine**	0.73	0.80	0.03	0.047
**Dispensable amino acids**	7.08	7.66	0.23	0.057
**Total amino acids**	14.36	15.46	0.36	0.040

1 Colostrum collected on the day of parturition, within 6 h of the birth of the first piglet.

Blood concentrations of glucose, urea, creatinine, IgG, IgA, and IGF-1 did not differ (*P *< 0.10) between treatments (Table 8). Insulin concentration showed a tendency to be greater (*P *= 0.077) in sows fed the Gln/Glu diet.

**Table 8.** Biochemical parameters in the serum of sows (d 20 of lactation) fed either a control diet or a diet supplemented with glutamine and glutamate (Gln/Glu) from d 86 of gestation to d 21 of lactation

Item	Control	Gln/Glu	SEM	*P*-Value
**Glucose, mg/dL**	45.8	42.0	1.5	0.134
**Urea, mg/dL**	29.4	30.1	1.0	0.756
**Creatinine, mg/dL**	1.88	1.81	0.04	0.498
**IgG, mg/dL**	753	780	24	0.794
**IgA, mg/dL**	8.14	8.05	0.51	0.938
**IGF-1, ng/ml**	107	100	7	0.217
**Insulin, microUI/ml**	3.61	5.42	0.71	0.077

IgG, Immunoglobulin G; IgA, Immunoglobulin A; IGF-1, Insulin-like growth factor I.

The bacterial load of *Escherichia coli* and *Clostridium perfringens* in fecal samples was not affected by dietary Gln/Glu supplementation (*P *> 0.10) (Table 9). For *Salmonella typhimurium*, the bacterial load was below the detection limit in the majority of samples, preventing statistical analysis for this variable.

**Table 9.** Fecal bacterial analyses of sows (d 20 of lactation) fed either a control diet or a diet supplemented with glutamine and glutamate (Gln/Glu) from d 86 of gestation to d 21 of lactation

Item^1^	Control	Gln/Glu	SEM	*P*-Value
**E. coli, copy^2^**	3.5×10^6^	5.5×10^6^	2.6	0.868
**C. perfringens, copy**	5.1×10^5^	2.5×10^5^	3.7	0.851

1 The bacterial load of Salmonella typhimurium was below the detection limit in the majority of samples, preventing statistical analysis for this variable.

2 Copy number/100 ng of DNA.

## Discussion

The present study evaluated the effects of glutamine and glutamate (Gln/Glu) supplementation during late gestation and lactation on sow performance, reproductive outcomes, colostrum composition, blood biochemical and immunological parameters, and fecal microbiota. The most notable effects of Gln/Glu supplementation were improved weaning weight, improved colostrum AA concentration, and reduced farrowing duration, while other performance parameters remained largely unaffected.

### Sow feed intake and weaning weight

Sows fed the Gln/Glu diet exhibited improved piglet weaning weight, which may be partially explained by a pull effect, in which greater milk yield stimulated higher ADFI during lactation (Strathe et al., 2017). This aligns with the roles of glutamine and glutamate as key functional AA during physiologically demanding periods such as lactation (Watford, 2015; Zhao and Kim, 2020). Both glutamine and glutamate serve as major oxidative fuels for rapidly proliferating cells, particularly enterocytes and immune cells (Wu, 1998; Stoll et al., 1999; Correia et al., 2023). Enhanced intestinal health mediated by glutamine and glutamate can reduce systemic inflammation and oxidative stress, conditions commonly exacerbated during lactation, potentially improving voluntary feed intake (Kim et al., 2006; Mateo et al., 2007). Moreover, glutamate serves as a precursor for glutathione, a major intracellular antioxidant, supporting oxidative balance during the high metabolic demands of lactation (Newsholme et al., 2003; Zhao and Kim, 2020).

In addition to gut health, both AA contribute to nitrogen transport and serve as precursors for other AA, nucleotides, and bioactive compounds (Wu, 1998; Correia et al., 2023). These functions are critical for supporting the metabolic pathways required for milk production. Although estimated milk yield was not statistically different between treatments, the higher weaning weight in the Gln/Glu group suggests improvements in milk composition or nutrient transfer to piglets. The increased concentrations of methionine, cystine, leucine, isoleucine, and tyrosine in colostrum support this interpretation, as these AA are essential for neonatal growth and immune development (Theil et al., 2014; Zhou et al., 2015). Although the improvement in piglet weaning weight was relatively small in absolute terms, such differences may still be relevant in commercial settings when scaled to herd level, as they can contribute to improved post-weaning growth.

Furthermore, the tendency for elevated insulin concentrations in the Gln/Glu group may reflect enhanced anabolic signaling, potentially driven by improved energy status mediated by glutamate metabolism in the intestinal-liver axis (Koketsu et al., 1998; Li et al., 2020). This improved metabolic efficiency could facilitate nutrient partitioning toward the mammary gland, supporting milk synthesis and contributing to improved litter performance.

### Reproductive outcomes

While total born, liveborn, and litter birth weight were greater in the Gln/Glu group, these differences are unlikely to be directly attributed to dietary supplementation. This is because the number of fetuses is biologically determined much earlier in gestation, as embryonic implantation is completed by day 35 (Theil et al., 2014), making it too late for a dietary treatment starting on d 86 to influence litter size. These differences are likely due to natural variability between treatment groups rather than a treatment effect. Additionally, piglet birth weight and within-litter birth weight variation were unaffected, indicating that fetal growth trajectories were similar between treatments.

### Farrowing process

The reduction in farrowing duration and shorter piglet intervals observed in sows supplemented with Gln/Glu is a noteworthy outcome that has been associated with lower risks of piglet hypoxia and improved piglet vitality (Oliviero et al., 2010; Peltoniemi et al., 2020). According to Oliviero et al. (2013), farrowing durations of 300 minutes or less can be considered an acceptable threshold. In our study, mean farrowing duration was 309 minutes in the Control group and 230 minutes in the Gln/Glu group, indicating that although both values were close to or below this level, supplementation further reduced farrowing time. While the direct effects of AA supplementation on the farrowing process are not widely documented, several physiological mechanisms could help explain this response. Farrowing is an energetically demanding event characterized by oxidative stress, inflammation, and progressive uterine muscle fatigue (Feyera et al., 2018; Peltoniemi et al., 2020). Both glutamine and glutamate play critical roles in antioxidant defense through their involvement in glutathione synthesis (Wu, 2009; Correia et al., 2023) and serve as substrates in energy metabolism via their entry into the TCA cycle (Yang et al., 2014; Rezaei et al., 2022). These functions are essential for maintaining cellular resilience and sustaining muscle contractility under metabolic stress. Therefore, it is plausible that improved oxidative balance and enhanced metabolic efficiency at the cellular level contributed to greater uterine efficiency during farrowing. Although further studies are needed to confirm this relationship, the present findings suggest that nutritional strategies targeting metabolic support in late gestation may positively influence farrowing performance.

### Colostrum composition and amino acid content

While Gln/Glu supplementation did not affect total colostrum dry matter, crude protein, ether extract, or lactose content, it significantly altered the AA content. The increase in specific AA observed in the colostrum of sows supplemented with Gln/Glu may reflect the central metabolic role of these AA in supporting mammary gland function and colostrum synthesis. This is particularly relevant for BCAA (leucine and isoleucine) and sulfur-containing AA (methionine and cystine), which are critical for protein synthesis, antioxidant defense, and immune function in neonates (Shen et al., 2014; Zhou et al., 2015; Gondret et al., 2021).

The increase BCAA, particularly leucine and isoleucine, observed in the colostrum of sows supplemented with Gln/Glu may be explained by the close metabolic interrelationship between glutamine, glutamate, and BCAA metabolism. Glutamate plays a central role as an amino group donor in transamination reactions required for the synthesis of BCAA in extrahepatic tissues, including the mammary gland (Li et al., 2009; Rezaei et al., 2022). This transamination process is crucial in tissues that lack full urea cycle functionality, such as the mammary gland, where nitrogen is conserved and redirected toward milk protein synthesis (Boyd et al., 1995; Holen et al., 2022).

In addition, the mammary gland exhibits high activity of branched-chain aminotransferases (BCAT), enzymes that depend on glutamate as a key nitrogen donor to convert branched-chain keto acids (BCKA) into BCAA (Rezaei et al., 2016). Therefore, enhanced glutamate availability from dietary supplementation likely increases substrate availability for this process, supporting higher synthesis or retention of BCAA in colostrum (Li et al., 2009; Li and Kim, 2024). Furthermore, both glutamine and glutamate serve as key anaplerotic substrates that feed into the TCA cycle, supplying the energy required for intensive AA and milk protein synthesis during early lactation (El-Kadi et al., 2009; Yang et al., 2014).

Beyond serving as building blocks, BCAA, especially leucine, are critical regulators of protein synthesis via activation of the mammalian target of rapamycin (mTOR) pathway in mammary epithelial cells (Li et al., 2009; Wang et al., 2018). This signaling enhances milk protein synthesis and possibly stimulates greater transport of AA into colostrum. The increased leucine and isoleucine observed in the current study align with this metabolic role and may partially explain the improved weaning weight of piglets, as BCAA are essential for muscle growth, protein accretion, and neonatal development (Holen et al., 2022).

A limitation of the present study is that only colostrum composition was analyzed, while milk composition during the lactation period was not determined. Therefore, it remains uncertain whether the improvements observed in colostrum AA content persisted throughout lactation. In addition, the greater number of liveborn piglets in the Gln/Glu group may have reduced individual piglet colostrum intake, which could have counteracted, at least in part, the beneficial effects of improved colostrum quality on piglet performance. Taken together, these factors highlight the need for future studies to evaluate milk composition across lactation and to account for differences in litter size when interpreting the impact of colostrum quality on piglet outcomes.

The higher levels of sulfur-containing AA (methionine and cystine) could be related to the interconnection between glutamine metabolism and the synthesis of other AA via transamination and amidation pathways (Newsholme et al., 2003). These sulfur AA are vital for antioxidant defense and protein synthesis, contributing to neonatal health and growth (Wu, 2009; Gondret et al., 2021). Moreover, glutamate itself serves not only as a substrate for milk protein synthesis but also plays a pivotal role as a signaling molecule in mammary epithelial cells (Rezaei et al., 2016). Its involvement in regulating mTOR signaling could stimulate protein synthesis and AA transport mechanisms, explaining the observed increases in essential AA.

The elevated tyrosine concentration observed in the colostrum of sows supplemented with Gln/Glu may be attributed to the close metabolic relationship between glutamate and aromatic AA synthesis. Tyrosine is synthesized from phenylalanine via phenylalanine hydroxylase; however, the availability of nitrogen donors—particularly glutamate—is critical for maintaining overall AA biosynthesis and turnover (Matthews, 2007). Glutamate plays a central role in transamination reactions, donating amino groups required for the synthesis of non-essential AA, including tyrosine (Wu, 1998). Moreover, tyrosine serves as a precursor for key bioactive compounds such as catecholamines and thyroid hormones, both of which are crucial for neonatal metabolic regulation, stress response, and thermoregulation in early life (Lossec et al., 1998; Bienboire-Frosini et al., 2023). Therefore, the improved maternal nitrogen economy and AA availability resulting from Gln/Glu supplementation may have facilitated greater tyrosine ­synthesis or retention in colostrum, potentially contributing to enhanced neonatal development.

### Blood biochemistry and immunological markers

The absence of significant changes in most blood biochemical and immunological parameters suggests that Gln/Glu supplementation did not disrupt systemic metabolic homeostasis. However, the tendency for higher insulin concentration may indicate improved nutrient-driven anabolic signaling during lactation, which can enhance nutrient partitioning toward milk production (Koketsu et al., 1998; Rezaei et al., 2016). Glutamate’s contribution to the TCA cycle and its known role as a metabolic signal could influence these endocrine responses, thereby supporting a more efficient utilization of energy at the cellular level (Wu, 1998; Yang et al., 2014). This suggests that even a small dietary addition of Gln/Glu may have a functional impact on the sow’s metabolic efficiency, further influencing her ability to support milk production.

### Fecal microbiota

The lack of differences in fecal *Escherichia coli* and *Clostridium perfringens* populations suggests that Gln/Glu supplementation did not alter the fecal bacterial composition in lactating sows. Glutamine and glutamate are known to support intestinal barrier function and modulate immune responses (Wu, 1998; Correia et al., 2023), which can indirectly influence gut microbial dynamics. A robust intestinal barrier, maintained by adequate nutrient supply to enterocytes, is critical in limiting bacterial translocation and adherence, thereby potentially restricting the proliferation of commensal or pathogenic bacteria within the gut lumen (Gomes et al., 2023; Abranches et al., 2025). However, the absence of changes in these specific bacterial populations indicates that any effects of Gln/Glu on intestinal health may have occurred independently of shifts in major pathogenic taxa.

### Conclusion

Supplementing sow diets with Gln/Glu improved piglet weaning weight, reduced farrowing duration, and improve the colostrum AA content, particularly concentrations of branched-chain, tyrosine, and sulfur-containing AA. While overall sow performance and most systemic biochemical parameters remained unchanged, the observed enhancements in colostrum composition and farrowing duration support the strategic inclusion of Gln/Glu as a nutritional intervention to improve reproductive efficiency and neonatal development. Further studies are warranted to elucidate the underlying mechanisms and to assess long-term effects on piglet health and performance.

## List of abbreviations

AA: amino acid
ADFI: average daily feed intake; BCAA, branched-chain amino acids
IgA: immunoglobulin A
IGF-1: Insulin-like growth factor I
IgG: immunoglobulin G
SID: Standardized ileal digestible
STTD: Standardized total tract digestible phosphorus
