# Correction to: Glutamine and glutamate supplementation in sow diets during late gestation and lactation reduces farrowing duration, improves colostrum amino acid content, and enhances piglet weaning weight

**DOI:** 10.1093/jas/skaf413

**Published:** 2025-12-15

This is a correction to: Maria Vitória S Sousa, Charles Kiefer, Lais Fernanda L Reis, Ana Gabrielli dos Santos Fagundes Euzebio, Luiza O Possa, Sung Woo Kim, Gabriel C Rocha, Glutamine and glutamate supplementation in sow diets during late gestation and lactation reduces farrowing duration, improves colostrum amino acid content, and enhances piglet weaning weight, *Journal of Animal Science*, Volume 103, 2025, skaf367, https://doi.org/10.1093/jas/skaf367.

The following changes have been made to the originally published manuscript. The name of the fourth author has been emended now to read: “Ana Gabrielli dos Santos Fagundes Euzebio”.

